# Machine learning identifies key cells and therapeutic targets during ferroptosis after spinal cord injury

**DOI:** 10.4103/NRR.NRR-D-24-00037

**Published:** 2024-07-29

**Authors:** Yigang Lv, Zhen Li, Lusen Shi, Huan Jian, Fan Yang, Jichuan Qiu, Chao Li, Peng Xiao, Wendong Ruan, Hao Li, Xueying Li, Shiqing Feng, Hengxing Zhou

**Affiliations:** 1 Department of Orthopedics, Tianjin Medical University General Hospital, International Science and Technology Cooperation Base of Spinal Cord Injury, Tianjin Key Laboratory of Spine and Spinal Cord, Tianjin, China; 2 Department of Orthopedics, Qilu Hospital of Shandong University, Cheeloo College of Medicine, Shandong University, Jinan, Shandong Province, China; 3 Key Laboratory Experimental Teratology of the Ministry of Education and Department of Physiology and Pathophysiology, School of Basic Medical Sciences, Shandong University, Jinan, Shandong Province, China; 4 Shandong University Center for Orthopedics, Advanced Medical Research Institute, Cheeloo College of Medicine, Shandong University, Jinan, Shandong Province, China; 5 State Key Laboratory of Crystal Materials, Shandong University, Jinan, Shandong Province, China; 6 Key Laboratory Experimental Teratology of the Ministry of Education, Department of Biochemistry and Molecular Biology, School of Basic Medical Sciences, Cheeloo College of Medicine, Shandong University, Jinan, Shandong Province, China; 7 Department of Orthopedics, The Second Hospital of Shandong University, Cheeloo College of Medicine, Shandong University, Jinan, Shandong Province, China; 8 Center for Reproductive Medicine, Shandong University, Jinan, Shandong Province, China

**Keywords:** bioinformatic analyses, bulk-RNA sequencing, cellular communication analysis, ferroptosis, machine learning analysis, neurological function, RNA velocity analysis, single-cell RNA sequencing, therapeutic drugs, transcription factor analysis

## Abstract

Ferroptosis, a type of cell death that mainly involves iron metabolism imbalance and lipid peroxidation, is strongly correlated with the phagocytic response caused by bleeding after spinal cord injury. Thus, in this study, bulk RNA sequencing data (GSE47681 and GSE5296) and single-cell RNA sequencing data (GSE162610) were acquired from gene expression databases. We then conducted differential analysis and immune infiltration analysis. *Atf3* and *Piezo1* were identified as key ferroptosis genes through random forest and least absolute shrinkage and selection operator algorithms. Further analysis of single-cell RNA sequencing data revealed a close relationship between ferroptosis and cell types such as macrophages/microglia and their intrinsic state transition processes. Differences in transcription factor regulation and intercellular communication networks were found in ferroptosis-related cells, confirming the high expression of *Atf3* and *Piezo1* in these cells. Molecular docking analysis confirmed that the proteins encoded by these genes can bind cycloheximide. In a mouse model of T8 spinal cord injury, low-dose cycloheximide treatment was found to improve neurological function, decrease levels of the pro-inflammatory cytokine inducible nitric oxide synthase, and increase levels of the anti-inflammatory cytokine arginase 1. Correspondingly, the expression of the ferroptosis-related gene *Gpx4* increased in macrophages/microglia, while the expression of *Acsl4* decreased. Our findings reveal the important role of ferroptosis in the treatment of spinal cord injury, identify the key cell types and genes involved in ferroptosis after spinal cord injury, and validate the efficacy of potential drug therapies, pointing to new directions in the treatment of spinal cord injury.

## Graphical abstract

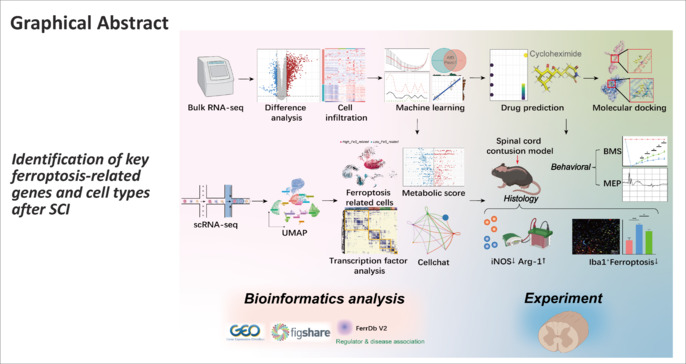

## Introduction

Spinal cord injury (SCI) is typically a devastating neurological condition caused by external trauma. It has high incidence and mortality (Hu et al., 2021), resulting in severe functional impairments of the nervous system (Fan et al., 2018). Current clinical treatment methods, including surgical decompression, pharmacotherapy, and rehabilitation training, are still unable to meet the challenges imposed by the complex pathological and physiological changes that lead to inflammatory damage and neurodegeneration (McDonald and Sadowsky, 2002).

A newly discovered cell death process known as ferroptosis has recently become the focus of significant research (Dixon et al., 2012). Correlations between ferroptosis and various disorders have been reported, including SCI, stroke, and neurodegenerative conditions (Do Van et al., 2016; Gu et al., 2024). After SCI, the primary injury causes local structural damage, and lipid peroxidation can result from secondary effects, such as bleeding, an inflammatory response, and oxidative stress (Li et al., 2022b). Furthermore, hemorrhage caused by the contusion results in iron accumulation within the injured spinal cord. SCI can be classified into three stages: the acute phase (< 2 days), the subacute phase (> 2 days to < 14 days), and the mid-chronic phase (> 14 days to < 6 months) (Ahuja et al., 2017). Ferroptosis predominantly manifests and progresses during the acute and subacute stages, influenced by iron ions and triggered by oxidative stress. The hallmark features of ferroptosis include the accumulation of lipid peroxides and depletion of glutathione (Cao et al., 2020). Acyl-coenzyme synthetase long-chain family member 4 (Acsl4), which is involved in lipid peroxidation, is upregulated following ferroptosis (Doll et al., 2017; Hassannia et al., 2019). Additionally, glutathione peroxidase 4 (Gpx4) and the glutamate/cystine antiporter system Xc^–^ (xCT/SLC7A11) play crucial roles in glutathione metabolism (Li et al., 2022c). Depletion of xCT and Gpx4 impairs cellular antioxidant capacity, leading to ferroptosis (Lei et al., 2019). Previous studies have found that the use of iron chelators such as deferoxamine (Yao et al., 2019), or antioxidants such as liproxstatin-1 (Fan et al., 2021) and edaravone (Pang et al., 2022), can inhibit ferroptosis and alleviate functional neurological impairments after SCI. Therefore, identifying potential therapeutic targets for intervention in post-SCI ferroptosis could provide new insights for the treatment of SCI.

In this study, we investigated the key regulatory genes and related cellular subtypes involved in ferroptosis by exploring gene expression profile data and single-cell transcriptome data from integrated SCI mouse models. First, we detected differentially expressed genes (DEGs) linked to the occurrence of ferroptosis at different time points after SCI, and predicted the key ferroptosis regulatory genes using machine learning methods. Next, by analyzing single-cell data, we determined the cellular subtypes related to ferroptosis and their intrinsic state transition processes. Furthermore, we analyzed the differences in transcription factor regulation and intercellular communication networks between the ferroptosis-associated cells. Finally, we predicted the effects of small molecule compounds on the key genes involved in ferroptosis.

## Methods

### Data collection

Four datasets, which were downloaded from the Gene Expression Omnibus (GEO, https://www.ncbi.nlm.nih.gov/geo/) and Figshare (https://figshare.com/), were used in this study. These included mRNA expression data from GSE47681 and GSE5296, transcriptome data from Figshare (https://doi.org/10.6084/m9.figshare.17702045) as a validation dataset for model construction, and single-cell transcriptome data from GSE162610 (Milich et al., 2021). After extracting data from the control group and samples 1, 3, and 7 days after thoracic SCI in mice, GSE47681 contained 34 samples (9 control and 25 SCI; Wu et al., 2013), GSE5296 contained 17 samples (8 control and 9 SCI; Guo et al., 2019), and Figshare contained 21 samples (6 control and 15 SCI; Li et al., 2022a). The ferroptosis-related gene dataset was obtained from FerrDb (http://www.zhounan.org/ferrdb/) and included a total of 394 related genes.

### Data processing and screening for key regulator genes

All statistical analyses were conducted using R software (v4.1.3, https://www.r-project.org/). The ComBat function in the sva package (v3.42.0, https://bioconductor.org/packages/release/bioc/html/sva.html) was used to eliminate batch effects after combining the large RNA sequencing datasets GSE5296 and GSE47681. To visualize the removal of batch effects, principal component analysis (PCA) was conducted using the FactoMineR package (v2.4, https://www.cran-e.com/package/FactoMineR) and factoextra (v1.0.7, https://cran.r-project.org/web/packages/factoextra/index.html) in R. The DEGs between the control and SCI groups were identified using the limma package (v3.46.0, https://bioinf.wehi.edu.au/limma/) with cutoff values of |log_2_(fold change)| > 1 and *P* < 0.05. The intersection between the ferroptosis-related genes and the DEGs was found using the UpSetR package (v1.4.0, https://cran.r-project.org/web/packages/UpSetR/index.html).

### Single-gene set variation analysis and immune infiltration analysis

The Gene Set Variation Analysis (GSVA) package (v1.42.0, https://github.com/rcastelo/GSVA) was used for GSVA (El Sehmawy et al., 2021). In this study, the gene set provided by FerrDb was used as the background gene set for GSVA. The GSVA function was used to calculate the ferroptosis scores for each sample. To analyze immune infiltration, we used the xCell package (v1.1.0, https://github.com/dviraran/xCell/) to compute the abundances of 44 immune cell types in each sample. Subsequently, based on feature genes of each cell type from the single-cell data, the immune cell abundance of 21 cell types in the samples was calculated by the ssGSEA algorithm. The cell infiltration abundance in the spinal cord tissue was visualized using the pheatmap package (v1.0.12, https://cran.r-project.org/web/packages/pheatmap/index.html). The Spearman correlation coefficients between the ferroptosis scores obtained by the GSVA algorithm and the immune cells were calculated using the ggcor package (v0.9.8.1, https://github.com/hannet91/ggcor).

### Machine learning to screen key regulator genes related to ferroptosis after spinal cord injury

To screen for key regulator genes related to ferroptosis after SCI and establish a regression model, we used the glmnet package (v4.1-3, https://www.rdocumentation.org/packages/glmnet/versions/4.1-3) to perform the least absolute shrinkage and selection operator (LASSO) algorithm for selection of the differentially expressed ferroptosis-related genes before and after SCI (Friedman et al., 2010). Subsequently, the random forest (RF) algorithm in the randomForest package (v4.7-1, https://www.rdocumentation.org/packages/randomForest/versions/4.7-1) was used to integrate multiple trees using ensemble learning to improve accuracy and narrow down the range of candidate biomarkers (Liaw and Wiener, 2002). The overlapping genes from the LASSO model and those with mean reduction Gini > 2 from the RF model were defined as key regulator genes for building the regression model. Subsequently, a regression model for the key ferroptosis-associated regulator genes was built using the ridge regression method with alpha set to 0 in the glmnet function. The ferroptosis risk score was calculated using the following formula: ferroptosis risk score = 0.7088 × Atf3 + 0.9349 × Piezo1 – 8.8020. The accuracy of the model was validated by receiver operating characteristic (ROC) curves in the GSE47681, GSE5296, and Figshare datasets using the pROC package (v1.18.0, https://www.rdocumentation.org/packages/pROC/versions/1.18.0).

### Single-cell RNA sequencing data analysis

Downstream analysis was performed on the single-cell transcriptome data from GSE162610 using the Seurat package (v4.1.0, https://www.rdocumentation.org/packages/Seurat/versions/4.1.0). To visualize clusters in a two-dimensional space, we employed the uniform manifold approximation and projection (UMAP) dimensionality reduction technique. The FindAllMarkers function and MAST method were used for differential expression analysis, with “logfc” set to 0.5, “min.pct” set to 0.5, and “min.diff.pct” set to 0.1. Quantification of the similarity between the single-cell data and bulk data was conducted using the SCISSOR package (v2.0.0, https://sunduanchen.github.io/Scissor/vignettes/Scissor_Tutorial.html; Sun et al., 2022). Pearson correlations were employed to determine the similarity between each pair of cells and the batch samples. A regression model was used to optimize the sample phenotype-related correlation matrix, and high-confidence selection of important cells with similar characteristics for a specific phenotype was performed. RNA velocity analysis of the single-cell data was performed using the velocity package (v0.6, https://www.rdocumentation.org/packages/velocyto.R/versions/0.6) in R and the scVelo package (v0.2.5, https://scvelo.readthedocs.io/en/stable/) in Python, followed by mapping cell types and phenotype-related cells from the single-cell data to display their internal transitions. Metabolic analysis of the single-cell data was performed using the Python package Compass (v0.9.10.2, https://yoseflab.github.io/Compass/tutorial.html; Wagner et al., 2021). The RcisTarget mouse database was downloaded from https://resources.aertslab.org/cistarget/ as a background file to construct the transcription factor regulatory network. A network was constructed using the SCENIC package (v1.3.1, https://rdrr.io/github/aertslab/SCENIC/). The AUCell algorithm was used to evaluate transcription factor activation, and regulatory submodules were identified based on connectivity specificity metrics. Finally, intercellular communication analysis was performed and visualized using the CellChat package (v1.6.1, https://rdrr.io/github/sqjin/CellChat/; Jin et al., 2021).

### Molecular docking analysis

Enrichment analysis was performed using drug–gene interaction data from the Drug Signatures Database (DSigDB, https://dsigdb.tanlab.org/DSigDBv1.0/) as the background gene set to find drugs that interact with the core genes. The two-dimensional molecular structures of the selected compounds were obtained from the PubChem database (https://pubchem.ncbi.nlm.nih.gov/). Protein structures were obtained from UniProt (https://www.uniprot.org/). AutoDock4 (v4.2.6, https://autodock.scripps.edu/download-autodock4/) software was used to perform flexible ligand docking simulations with a rigid protein and flexible small molecule in the PDBQT file. Finally, PyMOL (v2.2.0, https://pymol.org/) software was used to visualize the molecular docking results.

### Animals

The Animal Ethics Committee of Tianjin Medical University General Hospital (Tianjin, China; approval No. IRB2021-DW-74) approved the animal studies in this study. All experiments were designed and reported according to the Animal Research: Reporting of *In Vivo* Experiments (ARRIVE) guidelines (Percie du Sert et al., 2020). SPF (Beijing) Biotechnology Co. Ltd. (Beijing, China, license No. SCXK (Jing) 2019-0010) provided 8-week-old female C57BL/6J mice weighing 18–20 g, as the urethra of female mice is relatively short, giving a lower risk of injury during surgery. The animals were group-housed with free access to food and water. The animals were housed in alternating light and dark conditions for 12 hours. The temperature was kept between 20°C and 25°C, and the humidity was kept between 40% and 60%. The mice were randomly divided into three groups: the sham, SCI, and cycloheximide-treatment groups (*n* = 37 per group).

### Spinal cord injury model establishment and drug treatment

Animals were anesthetized by inhaled 4% isoflurane (RWD, R510-22, Shenzhen, China) before surgical procedures. When a satisfactory anesthetic effect was achieved, this was followed by 2% inhaled isoflurane, and skin preparation and disinfection were carried out, followed by incision of skin and muscle. The thoracic kyphosis at T7–T9 was exposed, and the lamina was cut with spring scissors to expose the T8 segment of the spinal cord. T8 laminectomy was performed, and the spinal cord was fully exposed. Spinous process fixators were used to fix the T7 and T9 spinous processes of mice. An NYU Impactor-III (WM Keck, New York, NY, USA) was then used to establish a moderate spinal cord contusion model (5 g × 12.5 mm) (LaPlaca et al., 2007). The muscle and skin were then sutured and sterilized again. The animal was placed on a heating pad at 37°C until the animal woke up. The bladder was manually emptied twice daily after SCI. Cefuroxime sodium (Servcorp, Nanjing, China; 6 mg/mL, dissolved in normal saline) was administered by intramuscular injection to prevent urinary tract infection. Mice in the sham group underwent laminotomy only.

For the cycloheximide-treated group, a 1 mg/kg dose of cycloheximide (Selleck, Houston, TX, USA) dissolved in 0.9% normal saline with 1% dimethylsulfoxide (DMSO; Solarbio, Beijing, China) was administered locally near the site of injury immediately after SCI based on previous research (Bey et al., 2017; Zhou et al., 2023). Mice in the SCI group were injected with the same dose of normal saline containing 1% DMSO, which was continued until the mice were ensuring minimal systemic absorption for targeted drug delivery. Mice were then anesthetized by inhaled 4% isoflurane, perfused with phosphate buffered saline in the heart, and treated according to the experimental strategy.

### Measurement of malondialdehyde levels and tissue iron

Tissue samples from the T8 spinal cord were collected and homogenized to prepare tissue homogenates. The homogenates were then centrifuged, and the supernatants were harvested. The malondialdehyde (MDA) content was determined using an MDA assay kit (Beyotime Biotechnology, Cat# S0131, Shanghai, China). An aliquot of the sample was treated with MDA detection working solution, followed by incubation in a boiling water bath and subsequent cooling and centrifugation. The optical density at 532 nm was measured, and the MDA content was calculated according to the manufacturer’s instructions. The tissue iron content was assessed using a tissue iron assay kit (Nanjing Jiancheng Bioengineering Institute, Cat# A039-2-1, Nanjing, China). Prepared samples were added to a reaction mixture containing reagents. After incubation in a boiling water bath, the samples were cooled and centrifuged to separate the supernatant. The absorbance of the supernatant at 520 nm was determined by microplate reader (Bio Tek, Winooski, VT, USA), and the tissue iron content was calculated according to the manufacturer’s instructions.

### Measurement of reactive oxygen species

Reactive oxygen species (ROS) levels in the T8 spinal cord tissue were quantified by dihydroethidium staining (Beyotime Biotechnology, Cat# S0063), in accordance with the manufacturer’s guidelines. Samples were incubated with dihydroethidium at 37°C for 30 minutes. After incubation, fluorescence microscopy was used to capture images of the stained samples. The percentage of the area that was dihydroethidium-positive was quantified by analysis with ImageJ software (v1.52a, National Institutes of Health, Bethesda, MD, USA; Schneider et al., 2012).

### Basso Mouse Scale

The recovery of hind limb motor behavior in the mice was assessed by the Basso Mouse Scale (BMS). Scoring was performed before injury, on days 1 and 7 after injury, and weekly until week 6 after injury, based on the BMS (Qu et al., 2021; Wang et al., 2021). The mice were adapted to a square open field (1 m in length and 1 m in width). Investigators were able to observe the movement behavior of the hind limbs of the mice. Mice were individually placed in the open field and observed by two investigators for 4 minutes. The observations were focused on monitoring hindlimb motor behavior, with scores ranging from 0 to 9 representing varying degrees of motor function. If there was no voluntary movement of the hind limbs, the score was 0, and the score for normal movement of the hind limbs was 9. A final score was determined based on multiple evaluation parameters, such as joint movement, foot sole position, step pattern, coordination, trunk stability, and tail control.

### Motor electrophysiology

Four weeks after SCI, mice were deeply anesthetized by inhaled 4% isoflurane. A stimulating electrode was inserted from the head of the mouse to the eye; a recording electrode was placed in the gastrocnemius muscle of the lower limbs of the mouse; and a ground electrode was placed on the skin of the back. Motor evoked potentials were analyzed using an electrophysiological device (Zhuhai Yiruikeji Co., Zhuhai, China) to assess the nerve injury repair. The potential latency and amplitude were subsequently analyzed.

### Hematoxylin and eosin staining

Mouse heart, liver, kidney, and lung were immersed in 4% paraformaldehyde solution for 24 hours, then dehydrated, cleared, and embedded in paraffin, and 5 µm paraffin sections were obtained using a paraffin microtome (Leica-Motorized, Wetzlar Germany). Paraffin sections were treated as follows: heated to 60°C for 4 hours, immersed in xylene I and II, sequentially immersed in 100%, 95%, 90%, 80%, and 70% alcohol solutions, rinsed with distilled water, stained with hematoxylin (Solarbio) for 2 minutes, differentiated with 1% hydrochloric acid, rinsed further with distilled water, and stained with 0.5% eosin staining solution (Solarbio) for 1 minute. The tissue sections were then dehydrated by placing them in gradient ethanol solutions (70%, 80%, 90%, and 100%) followed by immersion in xylene I and xylene II for 5 minutes each. Finally, the slides were sealed with neutral resin (Solarbio) and photographed using an inverted phase contrast microscope (Olympus BX53-M, Tokyo, Japan).

### Western blot assay

T8 spinal cord was lysed with radio immunoprecipitation assay lysate, then ground and centrifuged to obtain protein supernatant. The protein concentration was determined using the bicinchoninic acid (BCA) assay (Beyotime Biotechnology, Cat# P0010S). The target proteins were separated and transferred to a polyvinylidene fluoride (PVDF) membrane (Sigma, Darmstadt, Germany). After blocking with 5% bovine serum albumin in ddH_2_O, the PVDF membrane was incubated overnight at 4°C with primary antibody, then with secondary antibody at 24°C for 1 hour. Protein bands were visualized using ECL reagent (Beyotime Biotechnology), and images were captured using the ChemiDoc XRS System (Bio-Rad, Hercules, CA, USA). Quantitative analysis was performed using ImageJ software and normalized based on the ratio of target protein to α-tubulin. The following primary and secondary antibodies were used: rabbit anti-liver arginase 1 (Arg1; 1:1000; Abcam, Cambridge, UK, Cat# ab133543, RRID: AB_2943561), rabbit anti-inducible nitric oxide synthase (iNOS; 1:1000; Abcam, Cat# ab178945, RRID: AB_950738), rabbit anti-Gpx4 (1:1000; Abcam, Cat# ab125066, RRID: AB_2112414), rabbit anti-xCT (1:1000; ABclonal, Woburn, MA, USA, Cat# A13685, RRID: AB_2190860), mouse anti-α-tubulin (1:5000; Proteintech, Rosemont, IL, USA, Cat# HRP-66031, RRID: AB_11204167), HRP-labeled goat anti-rabbit IgG (1:1000; Beyotime, Cat# A0208, RRID: AB_3083707), and HRP-labeled goat anti-mouse IgG (1:1000; Beyotime, Cat# A0216, RRID: AB_2904020).

### Immunofluorescence staining

Spinal cord vertical segments, including the injury site, were collected and fixed overnight with 4% paraformaldehyde. After fixation, the spinal cord tissues were dehydrated in 30% sucrose solution for 72 hours, embedded in optimal cutting temperature (OCT) compound, and then cut into 10-µm-thick slices. After the sections were restored to room temperature, phosphate buffered saline was used to wash the OCT compound off the surface. Then, the sections were permeabilized and blocked prior to incubation with primary and secondary antibodies. Finally, the sections were incubated with DAPI (Beyotime Biotechnology, Cat# C1005) and capped with slides (CITOTEST Scientific, Jiangsu, China, Cat# 80340-0130) for observation. Fluorescence images were acquired using a fluorescence microscope (Leica-DMi85). ImageJ software was used for quantitative analysis of the Acsl4-, Gpx4-, and ionized calcium binding adapter molecule 1 (Iba1)-positive regions. The primary antibodies used were as follows: mouse anti-Iba1 (1:500; Cat# ab178846, RRID:AB_2924797, Abcam), rabbit anti-Gpx4 (1:400; Cell Signaling Technology, Danvers, MA, USA, Cat# 92880, RRID: AB_2877161), and rabbit anti-Acsl4 (1:400; Cell Signaling Technology, Cat# 92880, RRID:AB_2544613). The secondary antibodies were as follows: Alexa Fluor 555 goat anti-rabbit (1:500; Cat# ab 150078, RRID: AB_2544613, Abcam) and Alexa Fluor 488 goat anti-mouse (1:500; Cat# ab150113, RRID: AB_2936985, Abcam).

### Statistical analysis

The sample size was not predetermined using statistical methods; however, the number of samples in our study is comparable with those reported in previously published work (Li et al., 2023). No animals or data points were excluded from the analysis, which was conducted by researchers blinded to the experimental conditions. Statistical analyses were carried out using GraphPad Prism 8 (v8.0.2, GraphPad Software, Boston, MA, USA, https://www.graphpad.com/). For comparisons across multiple groups, one-way analysis of variance followed by Tukey’s *post hoc* test was used. Data are presented as mean ± standard deviation (SD). *P*-values < 0.05 were considered statistically significant.

## Results

### Identification of ferroptosis-associated differentially expressed genes following spinal cord injury and analysis of cellular infiltration in the microenvironment of the spinal cord

**Figure 1:**
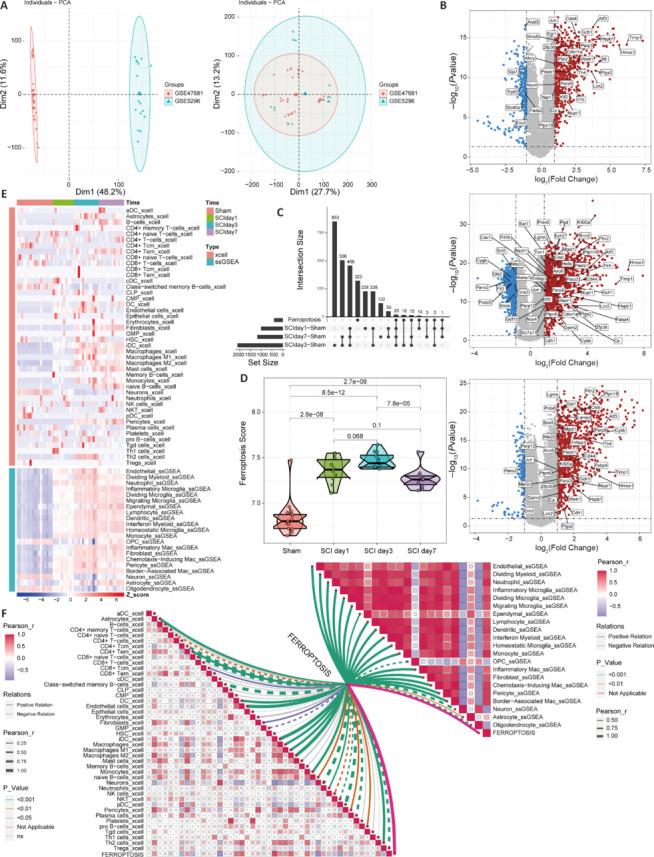
Identification of the differentially expressed genes associated with ferroptosis at different time points after SCI, and analysis of cell infiltration in the spinal cord microenvironment. (A) Principal component analysis of the GSE47681 and GSE5296 datasets before and after batch effect correction. (B) Analysis of differentially expressed genes 1, 3, and 7 days after SCI. The labels indicate the ferroptosis-related genes among the differentially expressed genes (*P*-value < 0.05 and |log_2_(fold change)| > 1). (C) Intersection of ferroptosis-related genes obtained from FerrDb with the differentially expressed genes in B. (D) Calculation of ferroptosis scores using the GSVA algorithm. (E) Evaluation of cell infiltration abundance from the transcriptomic data using the xCell and ssGSEA algorithms for single-cell differential gene analysis of the GSE162610 dataset. (F) Pearson correlations between the ferroptosis score and cell infiltration abundance calculated by the xCell algorithm (left), and correlations between the ferroptosis score and cell infiltration abundance calculated by the ssGSEA algorithm (right). GSVA: Gene Set Variation Analysis; SCI: spinal cord injury.

Here, our expanded sample size and effective integration of data from different sources allowed us to obtain robust research outcomes. We downloaded the GSE47681 and GSE5296 datasets and corrected for batch effects using sva. Principal component analysis demonstrated the effectiveness of this batch correction, as shown in **Figure 1A**. We then performed differential expression analysis to identify the DEGs 1, 3, and 7 days after SCI (**Figure 1B** and **Additional Table 1**). To study the association between SCI and ferroptosis, we downloaded ferroptosis-related genes from FerrDb and intersected them with the above DEGs (**Figure 1C**). We then calculated a ferroptosis score using GSVA software and found significant differences at the acute and subacute stages (**Figure 1D**). To associate the ferroptosis score with the cell types after SCI, we first evaluated the cell abundance in the transcriptome data using xCell software and the ssGSEA algorithm for the GSE162610 single-cell DEGs (**Figure 1E**). We then analyzed the correlation between the ferroptosis score and the abundance of infiltrating cells to determine the correlation between immune cells and the ferroptosis score, and the negative correlation between neurons, oligodendrocyte precursor cells (OPCs), and oligodendrocytes (**Figure 1F**). This demonstrates the potential regulatory role of ferroptosis in these cells.

**Additional Table 1:** Differential expression of ferroptosis-related genes at 1, 3, and 7 days after spinal cord injury

Group	Differentially expressed genes related to ferroptosis	Full names of the genes
Intersection genes	*Timp1*	Tissue inhibitor of metalloproteinases 1
	*Hmox1*	Heme oxygenase 1
	*Ptgs2*	Prostaglandin-endoperoxide synthase 2
	*Hspb1*	Heat shock protein family b (small) member 1
	*Atf3*	Activating transcription factor 3
	*Lcn2*	Lipocalin 2
	*Gch1*	GTP cyclohydrolase 1
	*Cd44*	CD44 molecule
	*Wwtr1*	WW domain containing transcription regulator 1
	*Tlr4*	Toll-like receptor 4
	*Parp3*	Poly(ADP-ribose) polymerase 3
	*Parp9*	Poly(ADP-ribose) polymerase 9
	*Zfp36*	ZFP36 ring finger protein
	*Nupr1*	Nuclear protein 1
	*Plin2*	Perilipin 2
	*Piezo1*	Piezo type mechanosensitive ion channel component 1
	*Cp*	Ceruloplasmin
	*Jun*	Jun proto-oncogene
	*Ano6*	Anoctamin 6
	*Ptpn18*	Protein tyrosine phosphatase, non-receptor type
1 d after SCI	*Timp1*	18 Tissue Inhibitor of Metalloproteinases 1
	*Hmox1*	Heme oxygenase 1
	*Il6*	Interleukin 6
	*Ptgs2*	Prostaglandin-endoperoxide synthase 2
	*Hspb1*	Heat shock protein family B (Small) member 1
	*Atf3*	Activating transcription factor 3
	*Lcn2*	Lipocalin 2
	*Gch1*	GTP cyclohydrolase 1
	*Cd44*	CD44 molecule
	*Wwtr1*	WW domain containing transcription regulator 1
	*Tlr4*	Toll-like receptor 4
	*Illb*	Interleukin 1 beta
	*Cdkn1a*	Cyclin-dependent kinase inhibitor 1A
	*Parp3*	Poly(ADP-ribose) polymerase 3
	*Parp9*	Poly(ADP-ribose) polymerase 9
	*Zfp36*	ZFP36 ring finger protein
	*Nupr1*	Nuclear protein 1
	*Plin2*	Perilipin 2
	*Piezo1*	Piezo type mechanosensitive ion channel component 1
	*Cp*	Ceruloplasmin
	*Jun*	Jun proto-oncogene
	*Ano6*	Anoctamin 6
	*Egr1*	Early growth response 1
	*Klf2*	Kruppel-like factor 2
	*Nox4*	NADPH oxidase 4
	*Yap1*	Yes1 associated transcriptional regulator
	*Ahcy*	Adenosylhomocysteinase
	*Ptpn18*	Protein tyrosine phosphatase, non-receptor type 18
	*Gja1*	Gap junction protein, alpha 1
	*Acsl3*	Acyl-CoA synthetase long chain family member 3
	*Fads2*	Fatty acid desaturase 2
	*Slc40a1*	Solute carrier family 40 member 1
	*Trp63*	Tumor protein p63
	*Eno3*	Enolase 3
3 d after SCI	*Hmox1*	Heme oxygenase 1
	*Timp1*	Tissue inhibitor of metalloproteinases 1
	*Kif20a*	Kinesin family member 20A
	*Atf3*	Activating transcription factor 3
	*Plin2*	Perilipin 2
	*Tlr4*	Toll-like receptor 4
	*Rrm2*	Ribonucleotide reductase regulatory subunit M2
	*Fabp4*	Fatty acid binding protein 4
	*Ptpn18*	Protein tyrosine phosphatase, non-receptor type 18
	*Ptgs2*	Prostaglandin-endoperoxide synthase 2
	*Lcn2*	Lipocalin 2
	*Nupr1*	Nuclear protein 1
	*Hspb1*	Heat shock protein family B (small) member 1
	*Gch1*	GTP cyclohydrolase 1
	*Piezo1*	Piezo type mechanosensitive ion channel component 1
	*Wwtr1*	WW domain containing transcription regulator 1
	*Parp9*	Poly(ADP-ribose) polymerase family member 9
	*Cd44*	CD44 molecule
	*Parp3*	Poly(ADP-ribose) polymerase 3
	*Cp*	Ceruloplasmin
	*Hells*	Helicase, lymphoid-specific
	*Cdkn1a*	Cyclin-dependent kinase inhibitor 1A
	*Ezh2*	Enhancer of zeste 2 polycomb repressive complex 2 subunit
	*Cybb*	Cytochrome b-245 beta chain
	*Zfp36*	ZFP36 ring finger protein
	*Ano6*	Anoctamin 6
	*Cav1*	Caveolin 1
	*Ctsb*	Cathepsin B
	*Prdx6*	Peroxiredoxin 6
	*Klf2*	Kruppel-like factor 2
	*Pgd*	Phosphogluconate dehydrogenase
	*Lgmn*	Legumain
	*Tnfaip3*	TNF alpha induced protein 3
	*Sat1*	Spermidine/spermine N1-acetyltransferase 1
	*Mgst1*	Microsomal glutathione S-transferase 1
	*Gstm2*	Glutathione S-transferase mu 2
	*Jun*	Jun proto-oncogene
	*Cdh1*	Cadherin 1
	*Txn1*	Thioredoxin 1
	*Rbms1*	RNA binding motif single stranded interacting protein 1
	*P4hb*	Prolyl 4-hydroxylase, beta polypeptide
	*Nfe2l2*	Nuclear factor, erythroid 2 like 2
	*Bach1*	BTB domain and CNC homolog 1
	*Slc7a11*	Solute carrier family 7 member 11
	*Ptpn6*	Protein tyrosine phosphatase, non-receptor type 6
	*Parpi2*	Poly(ADP-ribose) polymerase family member 12
	*Nox4*	NADPH oxidase 4
	*Gdf15*	Growth differentiation factor 15
	*Sirt3*	Sirtuin 3
	*Snca*	Synuclein, alpha
	*Gria3*	Glutamate ionotropic receptor AMPA type subunit 3
	*Fndc5*	Fibronectin type III domain containing 5
	*Cygb*	Cytoglobin
	*Flt3*	Fms related tyrosine kinase 3
	*Gls2*	Glutaminase 2
	*Panx2*	Pannexin 2
7 d after SCI	*Timp1*	Tissue inhibitor of metalloproteinases 1
	*Atf3*	Activating transcription factor 3
	*Hmox1*	Heme oxygenase 1
	*Ptpn18*	Protein tyrosine phosphatase, non-receptor type 18
	*Plin2*	Perilipin 2
	*Fabp4*	Fatty acid binding protein 4
	*Cybb*	Cytochrome b-245 beta chain
	*Tlr4*	Toll-like receptor 4
	*Parp9*	Poly(ADP-ribose) polymerase family member 9
	*Nupr1*	Nuclear protein 1
	*Hspb1*	Heat shock protein family B (small) member 1
	*Gch1*	GTP cyclohydrolase 1
	*Cd44*	CD44 molecule
	*Piezo1*	Piezo type mechanosensitive ion channel component 1
	*Ctsb*	Cathepsin B
	*Cdh1*	Cadherin 1
	*Tnfaip3*	TNF alpha induced protein 3
	*Parp3*	Poly(ADP-ribose) polymerase 3
	*Ptgs2*	Prostaglandin-endoperoxide synthase 2
	*Wwtr1*	WW domain containing transcription regulator 1
	*Lcn2*	Lipocalin 2
	*Kif20a*	Kinesin family member 20A
	*Sat1*	Spermine N1-acetyltransferase 1
	*Nfe2l2*	Nuclear factor erythroid 2-related factor 2
	*Cp*	Ceruloplasmin
	*Lgmn*	Legumain
	*Ano6*	Anoctamin 6
	*Parp12*	Poly(ADP-ribose) polymerase family member 12
	*Gstm2*	Glutathione S-transferase mu 2
	*Lamp2*	Lysosomal associated membrane protein 2
	*Mgst1*	Microsomal glutathione S-transferase 1
	*Ptpn6*	Protein tyrosine phosphatase non-receptor type 6
	*Zfp36*	ZFP36 ring finger protein
	*Snx5*	Sorting nexin 5
	*Prdx6*	Peroxiredoxin 6
	*Mef2c*	Myocyte enhancer factor 2C
	*Jun*	Jun proto-oncogene
	*Eno3*	Enolase 3
	*Panx2*	Pannexin 2

### Identification of key ferroptosis-related regulatory genes after spinal cord injury

**Figure 2:**
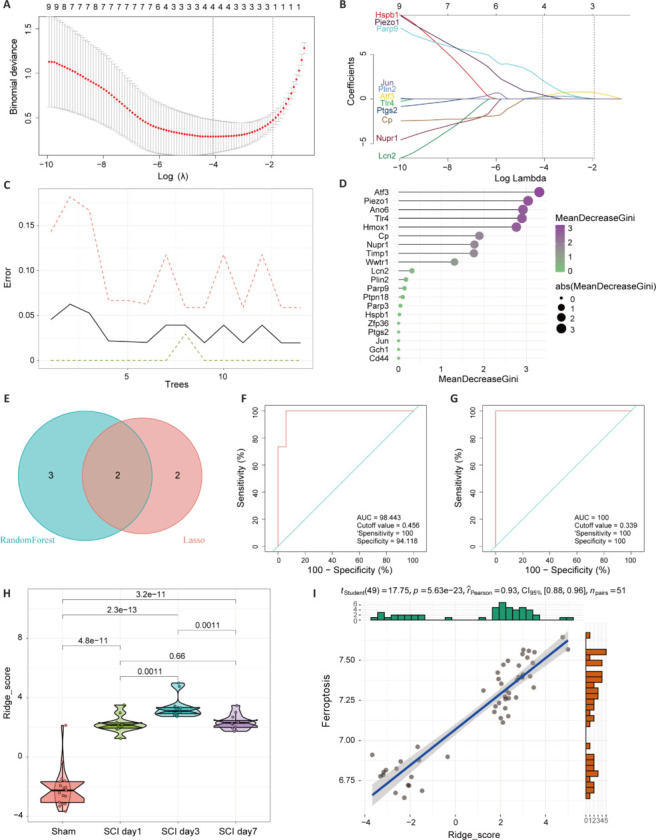
Selection of key ferroptosis-related regulatory genes after SCI. (A, B) LASSO regression analysis with the optimal λ value obtained through ten-fold cross-validation and adjustment of the penalty parameters. (C, D) Selection of feature genes with core scores greater than 2 using the RF algorithm. (E) Intersection of the feature genes selected by LASSO regression and RF. (F) Construction of a logistic regression model using ridge regression based on two key genes and evaluation of the accuracy using ROC curves. (G) Validation of the constructed ridge regression model using transcriptomic data from Figshare. (H) Distribution of the ridge regression model scores in the acute and subacute phases. (I) Pearson correlation between the scores from the ridge regression model and the ferroptosis score. LASSO: Least absolute shrinkage and selection operator; RF: random forest; ROC: receiver operating characteristic; SCI: spinal cord injury.

To screen for key regulatory genes related to ferroptosis after SCI, we used two different machine learning algorithms, LASSO and RF, to analyze the GSE47681 and GSE5296 datasets. The penalty parameters in the LASSO logistic regression model were adjusted using a ten-fold cross-validation approach. Through this process, four specific features associated with ferroptosis after SCI were selected by the regression model (**Figure 2A** and **B**). Then, the data from the RF algorithm were filtered to obtain five feature genes with a core score greater than 2 points (**Figure 2C** and **D**). In the subsequent analysis, the marker genes obtained based on the two algorithms were intersected to obtain two marker genes, Atf3 and Piezo1 (**Figure 2E**). Using the R package glmnet, a logistic regression model was constructed based on these two key genes using ridge regression. The accuracy of the regression model was assessed using ROC curve analysis, yielding an area under the ROC curve (AUC) value of 0.98 (**Figure 2F**). Based on the cutoff value determined from the ROC curve, we classified the samples into two groups: one with a high ferroptosis score and one with a low ferroptosis score. These data were then validated with the transcriptome data from Figshare (**Figure 2G**). We found significant differences in the ferroptosis prognostic model at the acute and subacute stages (**Figure 2H**), as well as a good correlation with the ferroptosis score (**Figure 2I**). This indicates that, to some extent, this model can represent ferroptosis after SCI.

### Identification of ferroptosis-related cells by single-cell RNA sequencing analysis

**Figure 3:**
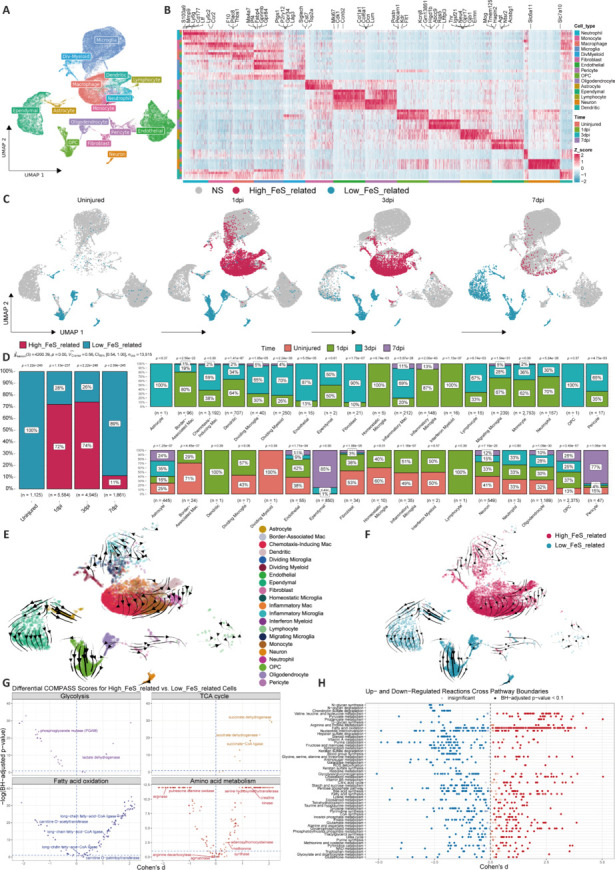
Identification of the types of cells related to ferroptosis after SCI. (A) Cell types were determined from the single-cell sequencing data at 1, 3, and 7 days after SCI and the control group. These cells were classified into 15 types, including neutrophils, monocytes, macrophages, microglia, fibroblasts, endothelial cells, pericytes, OPCs, neurons, and ependymal cells. (B) Display of the DEGs and marker genes in the 15 main cell types. The colors in the top and side bars represent specific cell clusters. (C) Integration of the sequencing data from GSE47681 and GSE5296 with the corresponding ferroptosis subarray information using SCISSOR software to analyze cells associated with different ferroptosis subarrays. (D) Bar plots showing the proportions of cells related to certain ferroptosis scores at different time points (left) and the proportions of cells positively correlated (top right) or negatively correlated (bottom right) with ferroptosis scores among the different cell types. (E) Inference of cell differentiation trajectories using scVelo. (F) Mapping of the cells related to high and low ferroptosis scores onto the cell differentiation trajectory. (G, H) Comparison of metabolic pathways between these two distinct ferroptosis-associated cell populations using Compass software. DEGs: Differentially expressed genes; OPC: oligodendrocyte precursor cell; SCI: spinal cord injury.

Next, we aimed to elucidate the cell types associated with ferroptosis after SCI. From the GSE162610 single-cell sequencing data 1, 3, and 7 days post-injury (dpi) of the SCI and control groups, cells were divided into 10 cell types, including neutrophils, monocytes, macrophages, microglia, fibroblasts, endothelial cells, pericytes, OPCs, neurons, and ependymal cells (**Figure 3A** and **B**). We used the SCISSOR algorithm to combine the GSE47681 and GSE5296 sequencing data with the corresponding ferroptosis score data after SCI to elucidate which cells are associated with the different ferroptosis score groups (**Figure 3C**). Consistent with the higher ferroptosis score in the bulk sequencing data at 3 dpi, more cells with high ferroptosis scores were found in the single-cell sequencing data at 3 dpi (**Figure 3D**, left), and inflammatory microglia and chemotactic macrophages were the main cells showing high ferroptosis scores (**Figure 3D**, right). The cell differentiation trajectories were then inferred using scVelo (**Figure 3E**). A transition from cells with a low ferroptosis score to those with a high ferroptosis score was found, specifically the transitions from resting microglia to inflammatory microglia and border-associated macrophages to chemotactic macrophages (**Figure 3F**). The differences in metabolic pathways between these two different ferroptosis-associated cell groups were then compared using Compass software, and increased lipid metabolism processes were found in the high-score group of cells (**Figure 3G** and **H**).

### Differential transcription factor regulation and the ferroptosis-associated cell communication network

**Figure 4:**
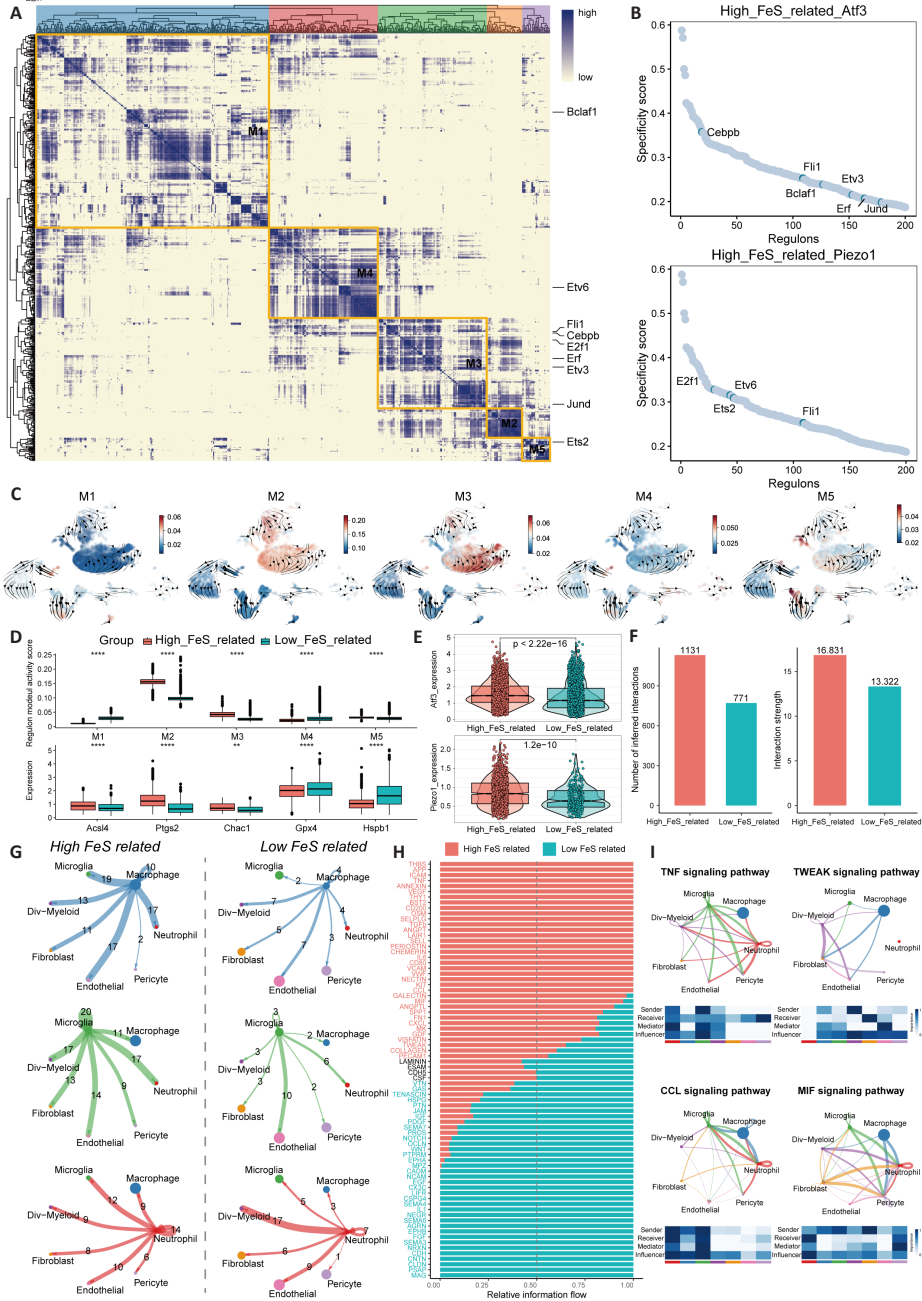
Analysis of differences in transcription factor regulation and the intercellular communication network in ferroptosis-associated cells. (A) Correlation matrix constructed based on the RAS of each regulon. The regulons were clustered into five distinct modules (M1, M2, M3, M4, and M5), with the regulons within each module exhibiting similar transcriptional activities. The labels indicate the transcription factors regulating *Atf3* and *Piezo1*, which are primarily concentrated in modules M2, M3, and M5. (B) Ranking of the activity scores of all transcription factors in high-ferroptosis cells. The labels indicate the regulation of the transcription factors *Atf3* and *Piezo1*. (C) Mapping of the RASs of the five modules onto RNA velocity to validate their associations with different ferroptosis subgroups. (D) Differences in the distributions of the module scores (top) and ferroptosis-related marker genes (bottom) between ferroptosis-positive and ferroptosis-negative cells. (E) Differences in the expression levels of *Atf3* and *Piezo1* between ferroptosis-positive and ferroptosis-negative cells. (F) Cell‒cell interactions inferred using CellChat; the number and strength of the cell communications associated with the ferroptosis scores are displayed. (G) Circos plot depicting the presumed ligand‒receptor interactions between macrophages, astrocytes, neutrophils, and other cells in ferroptosis-positive and ferroptosis-negative cells. (H) Overall differences in information flow in the inferred network between ferroptosis-positive and ferroptosis-negative cells. (I) Cell communication in various cell types involving TNF, the TWEAK pathway, CCL chemokines, and the macrophage migration inhibitory factor signaling pathway in ferroptosis-positive cells. Atf3: Activating transcription factor 3; CCL: chemoattractant cytokine ligand; Piezo1: Piezo type mechanosensitive ion channel component 1; RAS: regulatory activity score; SCI: spinal cord injury; TNF: tumor necrosis factor; TWEAK: TNF-like weak inducer of apoptosis.

Next, we evaluated the differences in transcription factor activation in different ferroptosis subgroups (**Figure 4A**). Transcription factor activity refers to the regulatory capacity of transcription factors on genes within the gene regulatory network or the level of activity they exhibit in gene regulation. Building upon previous work (Suo et al., 2018), we employed SCENIC software to assess the potential regulatory genes of each regulon and calculated regulon activity scores based on the expression of their target genes. Additionally, we clustered the regulons into five different modules (M1, M2, M3, M4, and M5). We found that the transcription factor modules regulating *Atf3* and *Piezo1* were mainly modules M2, M3, and M5. **Figure 4B** shows the activity score rankings of the transcription factors regulating *Atf3* and *Piezo1* in the high-ferroptosis cells. We also performed RNA velocity analysis and mapped the regulon activity scores of the five modules to determine their relationship with different ferroptosis subgroups (**Figure 4C**). The M2, M3, and M5 modules were mainly active in the high-ferroptosis group, while the M1 and M4 modules were mainly active in the low-ferroptosis group (**Figure 4D**, top). The ferroptosis-related marker genes in the high-score group were also upregulated (**Figure 4D**, bottom). Moreover, we confirmed the differences in the *Atf3* and *Piezo1* expression levels between the two groups (**Figure 4E**). Next, we used CellChat to infer the interactions between cells related to the ferroptosis score and found that there was more cell communication, in terms of both quantity and intensity, in the high-score group (**Figure 4F**). Changes in communication in macrophages, microglia, and neutrophils, which were mainly concentrated in the high-ferroptosis group, may be important (**Figure 4G**). In addition, the tumor necrosis factor (TNF), TNF-like weak inducer of apoptosis (TWEAK), chemoattractant cytokine ligand (CCL) chemokine, and macrophage migration inhibitory factor signaling pathways were upregulated in the high-score group (**Figure 4H** and **I**).

### Validation of the key ferroptosis genes and drug prediction

**Figure 5:**
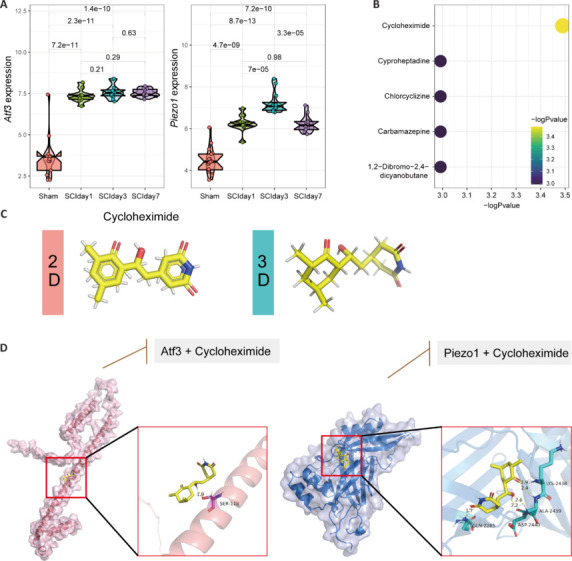
Drug screening of the core ferroptosis genes after intervention in SCI. (A) Comparison of the differences in the expression levels of *Atf3* and *Piezo1* at different time points. (B) Prediction of small molecular compounds that may interact with the proteins encoded by the core genes involved in ferroptosis based on the results from DSigDB. (C) Two-dimensional (left) and three-dimensional (right) structures of cycloheximide. (D) Prediction of the sites at which cycloheximide binds with the ATF3 and PIEZO1 proteins using AutoDock software. Atf3: Activating transcription factor 3; Piezo1: Piezo type mechanosensitive ion channel component 1; SCI: spinal cord injury.

To screen for drugs that can intervene in the interactions between core ferroptosis genes after SCI, we compared the differences in the expression levels of *Atf3* and *Piezo1* at different time points (**Figure 5A**). Subsequently, based on analysis of DSigDB, we hypothesized that cycloheximide could serve as a small-molecule compound that binds to the identified ferroptosis hub genes (**Figure 5B** and **C**). We also successfully predicted the sites at which cycloheximide binds with the ATF3 and PIEZO1 proteins using AutoDock software (**Figure 5D**).

### *In vivo* functional and histological drug validation

**Figure 6:**
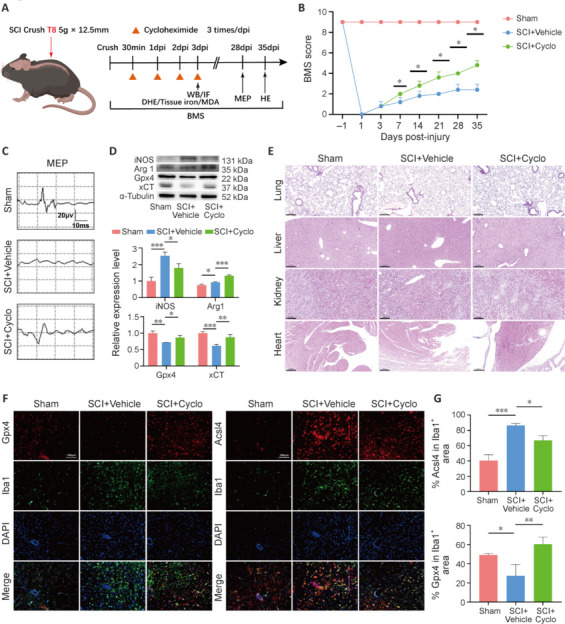
*In vivo* functional and histological evaluations of the therapeutic effect of cycloheximide after SCI. (A) Schematic of the establishment of a spinal cord contusion model via exposure of the T8 segment, followed by drug intervention and histological/functional assessments at the indicated time points. (B) BMS locomotor function scores for 35 dpi in the sham, SCI, and cycloheximide (Cyclo) groups (*n* = 4). (C) Improvement in MEP 28 dpi in the cycloheximide group (*n* = 4). (D) Analysis of tissue samples 2 weeks after SCI revealed changes in the iNOS, Arg1, Gpx4, and xCT levels in the cycloheximide group compared with the SCI group (*n* = 3). (E) HE-stained sections of the heart, kidney, liver, and lung from the sham, SCI, and cycloheximide-treated mice 35 dpi, showing no significant morphological changes (*n* = 3). Scale bars: 200 µm. (F) Immunofluorescence staining of microglia/macrophages showing Iba1 (green, Alexa Fluor 488), Gpx4 (left, red, Alexa Fluor 555), Acsl4 (right, red, Alexa Fluor 555), and the nucleus (blue) after cycloheximide treatment. After cycloheximide treatment, the amount of Gpx4 in Iba1-positive cells increased, while the amount of ACSL4 decreased. Scale bars: 100 µm. (G) Quantitative analysis of Gpx4 and Acsl4 in microglia/macrophages (*n* = 3). Data are expressed as mean ± SD. *P < 0.05, **P < 0.01, ***P < 0.001. Acsl4: Acyl-coenzyme synthetase long chain family member 4; ARG1: arginase-1; BMS: Basso Mouse Scale; dpi: days post-injury; Gpx4: glutathione peroxidase 4; HE: hematoxylin and eosin; Iba1: ionized calcium binding adapter molecule 1; IF: immunofluorescence; iNOS: inducible nitric oxide synthase; MEP: motor evoked potential; SCI: spinal cord injury; WB: western blot; xCT: glutamate/cystine antiporter system Xc^–^.

We successfully constructed a spinal cord contusion animal model at the T8 level by exposing the spinal cord at the T8 segment (**Figure 6A**). The cycloheximide group showed an improvement in BMS locomotor test scores by 28 dpi, although there was no significant improvement observed with cycloheximide during the first 3 days following SCI (**Figure 6B**). Furthermore, motor evoked potentials were improved by 28 dpi in the cycloheximide group (**Figure 6C**). At the 2-week post-SCI follow-up, analysis of tissue samples revealed that the cycloheximide group showed significantly lower levels of inducible nitric oxide synthase (iNOS) and higher levels of arginase 1 (Arg1) compared with the SCI group. The expression levels of the ferroptosis marker genes *Gpx4* and *xCT* (*Slc7a11*) were upregulated (*P* < 0.05; **Figure 6D**). Notably, histological staining using hematoxylin and eosin did not show significant morphological changes in the heart, kidneys, liver, or lungs of mice with SCI after 6 weeks of cycloheximide treatment (**Figure 6E**). After cycloheximide treatment, Gpx4 showed enhanced red fluorescence, while Acsl4 showed weaker red fluorescence (**Figure 6F** and **G**). Additionally, to further ascertain whether cycloheximide mitigated ferroptosis post-SCI, we assessed the levels of ROS, iron accumulation, and lipid peroxidation within the spinal cord tissues. We noted that cycloheximide ameliorated the ROS levels in microglia/macrophages post-SCI (**Additional Figure 1A** and **B**), and reduced the total iron content and MDA levels in spinal cord tissues (**Additional Figure 1C** and **D**). In conclusion, these findings provide evidence supporting the potential of cycloheximide to reduce ferroptosis, suppress the production of inflammatory factors, and facilitate functional recovery in the context of SCI.

## Discussion

Ferroptosis, a recently identified form of programmed cell death characterized by its unique iron-dependent mechanism (Stockwell et al., 2017), has attracted considerable recent attention. This process plays a crucial role in various central nervous system diseases, including traumatic brain injury, stroke, and neurodegenerative disorders such as Alzheimer’s disease (Hambright et al., 2017; Kenny et al., 2019; Dong et al., 2020; Qu et al., 2021; Zhao et al., 2021). While early research focused on the ferroptosis of neurons, this study, combining machine learning techniques with bulk and single-cell sequencing data, reveals the dominant role of microglia/macrophages in ferroptosis following SCI for the first time. It identifies *Atf3* and *Piezo1* as potential regulatory genes. Moreover, we successfully identify cycloheximide as a drug that can potentially intervene in the ferroptosis of microglia/macrophages, improving the immune microenvironment and promoting recovery of neurological functions, and offering new strategies for the treatment of central nervous system injuries.

We aimed to investigate the regulatory mechanisms of ferroptosis during the acute and subacute phases of SCI. After the GSVA algorithm was used to identify ferroptosis-related genes, we demonstrated that ferroptosis regulation is critical in the first 3 days after SCI, with a decrease in the effects of ferroptosis regulation occurring after 3 days. Furthermore, we selected DEGs related to ferroptosis as potential biomarkers. We focused on DEGs that exhibited differences in expression at different time points after SCI, including previously reported drivers of ferroptosis, such as *Atf3*, *Piezo1*, *Tlr4*, *Timp1*, *Hmox1*, *Wwtr1*, and *Ano6*. ATF3 is a member of the ATF/CREB protein family. A recent study has suggested that ATF3 can induce ferroptosis by inhibiting the NRF2/KEAP1/xCT signaling pathway (Fu et al., 2021). PIEZO1 is a mechanically activated nonselective cation channel (Coste et al., 2010) expressed in macrophages, where it can disrupt the levels of the iron-regulating factor hepcidin, leading to iron overload (Ma et al., 2021). TLR4 can initiate activation of the inflammatory pathway. Previous studies have shown that oxygen-glucose deprivation induces TLR4 upregulation, an increase in p53 activation, and the loss of xCT/SLC7A11 and Gpx4, resulting in neuronal ferroptosis (Zhu et al., 2021). In addition, TIMP1 (tissue inhibitor of metalloproteinase 1) overexpression, mediated by knocking out miR-30b-5p, enhances the activation of transferrin receptor protein 1 (TFR-1), ultimately leading to ferroptosis in cardiac microvascular endothelial cells (Shi et al., 2022). Heme oxygenase is a key enzyme in the breakdown of heme, particularly the degradation of heme derived from red blood cells engulfed by macrophages. Heme is initially metabolized by heme oxygenase, producing iron, carbon monoxide, and biliverdin. Consequently, this process further enhances ROS generation and lipid peroxidation. Furthermore, iron can be released into the surrounding environment through ferroportin, leading to an increase in the iron content in macrophages and the tissue microenvironment (Yang et al., 2022). *Hmox1* is associated with the polarization of M1-type microglia/macrophages after SCI (Xuan et al., 2023). WWTR1 (WW domain-containing transcription regulator 1) regulates erastin-induced ferroptosis by inducing angiopoietin like 4 (ANGPTL4), thereby activating nicotinamide adenine dinucleotide phosphate hydrogen oxidase 2 (NOX2) and leading to ferroptosis (Yang et al., 2020). Anoctamin 6 (ANO6) is a Ca^2+^-activated chloride/nonselective ion channel that increases the exposure of phosphatidylserine and disrupts membrane stability (Martins et al., 2011; Ousingsawat et al., 2015). During macrophage ferroptosis caused by erastin and RSL3, phospholipid peroxidation occurs on the cell membrane, activating the ANO6 channel and ultimately leading to cell death (Ousingsawat et al., 2019). The identification of ferroptosis remains a significant priority in this field. Currently, there are no specific molecular markers or staining methods that can accurately detect ferroptosis (Gao et al., 2022). In this study, we used two machine learning algorithms to explore the core molecular markers of ferroptosis in post-SCI tissues. Through this approach, we identified the core genes *Atf3* and *Piezo1*. Furthermore, we built a ridge regression model by integrating the expression levels of these genes. The overall aim was to determine the key regulatory period following SCI by combining multiple ferroptosis-related biomarkers.

Distinguishing between activated microglia and infiltrating macrophages after SCI is challenging, as both exacerbate inflammatory damage through the release of proteases, ROS, and inflammatory factors (Hellenbrand et al., 2021). Macrophages are vital immune cells that exist in two distinct phenotypes: classically activated (M1) and alternatively activated (M2) macrophages. The phagocytic activity of macrophages, along with the generation of ROS and the release of inflammatory cytokines including TNF-α, IL-1β, and IL-6, collectively influence the activities of enzymes involved in iron, lipid, and amino acid metabolism. These factors have implications for the cellular status of tissue cells and the macrophages themselves (Yang et al., 2022). Our study showed that genes related to the CCL signaling pathway, which is associated with macrophage recruitment, were highly expressed in cell types with higher ferroptosis scores. Activation of the CCL signaling pathway in ferroptotic cells can potentially be initiated by activation of the TNF signaling pathway (Djudjaj et al., 2017; Lv et al., 2018; Wang et al., 2021). Based on the RNA velocity data, the activation of the transcription factor regulon activity score in modules M2, M3, and M5 suggests that these modules are activated in the high-ferroptosis group, while modules M1 and M4 are activated in the low-ferroptosis group. These findings suggest that the transcription factors M2, M3, and M5 may play a role in regulating ferroptosis, the associated inflammatory pathways, and macrophage phenotype transitions following SCI.

Targeting ferroptosis is of great importance in the repair of SCI. We predicted candidate pharmacological compounds through the two core genes using DSigDB. Drug discovery is a crucial component of the identification of potential new drugs from existing molecular databases. This approach is imperative for the treatment of human diseases. Drug discovery involves two key components: proteins (targets) and small molecules (ligands), which form protein‒ligand interactions. In this study, cycloheximide was demonstrated to act as a ligand that can inhibit the activity of the ATF3 and PIEZO1 proteins. Cycloheximide primarily exerts its pharmacological effects by forming polar covalent bonds with Ser118 of the ATF3 protein and with Ala2439, Asp2440, Lys2438, and Gln2285 of the PIEZO1 protein. However, validation through liquid chromatography and mass spectrometry analyses is needed to confirm the accuracy of the docking simulation results.

This study also has several limitations. First, in bulk RNA sequencing data analysis, it is important to address potential batch effects arising from different datasets, even after preprocessing the data using the sva package. Further experimental validation is needed to assess the correlation between microglia and ferroptosis, by the use of single-cell RNA sequencing to deconvolute cell types and evaluate the abundance of cell infiltration in the bulk RNA sequencing data. Additionally, it should be noted that in AutoDock, which allows docking of a single receptor‒ligand pair, the identification of potential binding interactions between cycloheximide and genes other than the hub genes in macrophages/microglia may be limited, although these other interactions may contribute to its pharmacological effects. Cycloheximide, an antifungal antibiotic, inhibits protein synthesis but carries toxicity that restricts clinical use. Recent studies have suggested its role in mitigating ferroptosis (Rashad et al., 2022), offering therapeutic promise for ischemic hypoxic brain injury (Park et al., 2006a, b). To minimize its toxicity, our study used local injections to reduce the systemic side effects, informing future applications requiring precise dosing and administration techniques. Advancing the clinical utility of cycloheximide may involve the development of safer derivatives (Edlich et al., 2006) that retain the anti-ferroptotic properties. Ongoing research is needed to refine the clinical use of cycloheximide. Moreover, exploring the role of the hub genes (*Atf3* and *Piezo1*) in SCI by gene knockdown is an avenue we intend to investigate.

In summary, this study used multiple datasets and analysis methods to investigate key regulatory genes and cell types associated with ferroptosis at different time points during the acute and subacute phases of SCI. First, we downloaded four datasets, including mRNA expression data and transcriptomic data, from GEO and Figshare. These datasets were used as the validation dataset for model construction and for single-cell transcriptomic analysis. After data processing and filtering, we identified DEGs using the statistical analysis tool R, and conducted intersection analysis with the genes associated with ferroptosis. Our results confirmed that the period from 1 to 3 days after SCI is a critical phase in ferroptosis progression. We assessed the correlation between ferroptosis scores and immune cells through single-gene set variation analysis and immune infiltration analysis. Next, we employed the LASSO and RF algorithms to select key regulatory genes associated with ferroptosis and constructed a regression model. This model calculated the ferroptosis risk score using these key genes and validated the accuracy of the model through ROC analysis. Additionally, by analyzing single-cell RNA sequencing data, we classified the cell types associated with ferroptosis and further explored the single-cell data using various analytical tools, including UMAP dimensionality reduction visualization, differential expression analysis, RNA velocity analysis, and metabolic analysis. Finally, molecular docking was conducted to discover drugs that can interact with the core genes. Overall, this study revealed key regulatory genes and cell types associated with ferroptosis after SCI, along with their correlation with immune infiltration, by analysis at multiple levels. These findings contribute to a more profound understanding of the mechanisms of ferroptosis in the pathological process of SCI and provide an important foundation for the development of related therapeutic strategies. However, many challenges still exist, and future research directions need to be explored to improve and advance the development of SCI treatment.

## Additional files:

***Additional Figure 1:*** *Changes in ferroptosis-related markers after cycloheximide treatment in SCI mice.*

Additional Figure 1 Changes in ferroptosis-related markers after cycloheximide treatment in SCI mice.
(A) Fluorescent staining of DHE (red) in microglia/macrophages (Ibal-positive cells, green, Alexa Fluor 488) and nucleus (blue) after treatment with cycloheximide. After cycloheximide treatment, the red fluorescence intensity of DHE in Ibal positive cells decreased. Scale bars: 100 μm. (B) Quantitative analysis of DHE in microglia/macrophages (*n* = 3). (C) Iron levels in spinal cord tissue post-cycloheximide treatment (*n* = 7). (D) MDA levels in spinal cord tissue after cycloheximide treatment (*n* = 7). Data are expressed as mean ± SD (*n* = 3). *P < 0.05, ***P < 0.001, ****P < 0.0001 (one-way analysis of variance with Tukey’s post hoc test). DHE: Dihydroethidium; Ibal: ionized calcium binding adapter molecule 1; MDA: malondialdehyde; SCI: spinal cord injury.

***Additional Table 1:*** *Differential expression of ferroptosis-related genes at 1, 3, and 7 days after spinal cord injury.*

## Data Availability

*All data analyzed during this study were sourced from Gene Expression Omnibus (GEO) (Nos. GSE47681 and GSE5296), and Figshare (10.6084/m9.figshare.17702045)*.
