# Supplementary figures and images for: Machine learning identifies key cells and therapeutic targets during ferroptosis after spinal cord injury
Source: Neural Regen Res. 2024 Jul 29;21(6):2495–505. doi: 10.4103/NRR.NRR-D-24-00037 (PMC13211781; doi:10.4103/NRR.NRR-D-24-00037)

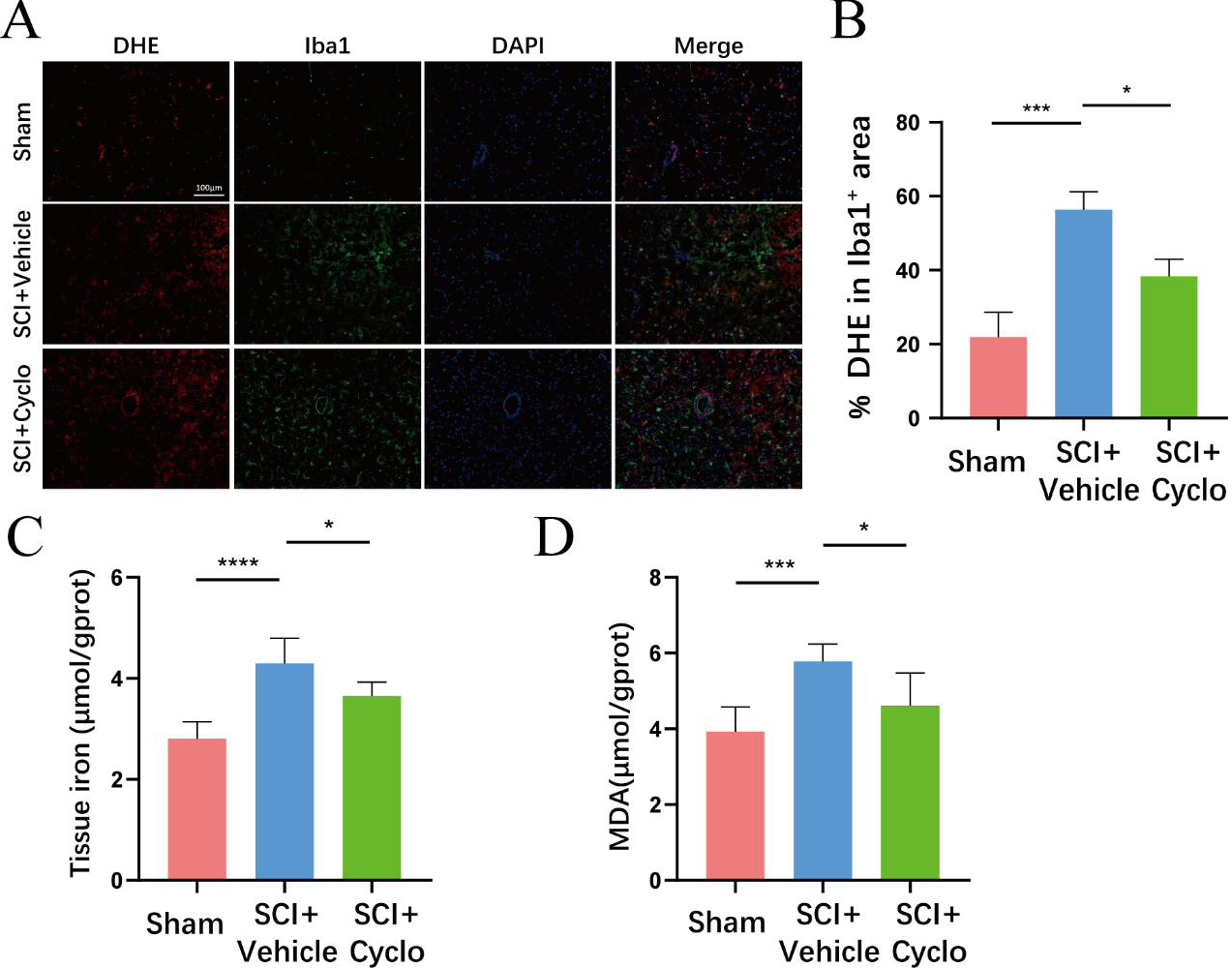

Supplement: Supplementary file 1 [file NRR-21-2495_Suppl1.tif]
